# Mutational Profile Evaluates Response and Survival to First‐Line Chemotherapy in Lung Cancer

**DOI:** 10.1002/advs.202003263

**Published:** 2020-12-30

**Authors:** Yayi He, Lele Song, Hao Wang, Peixin Chen, Yu Liu, Hui Sun, Xiaobin Li, Shiying Dang, Guifeng Liu, Xinyi Liu, Shifu Chen, Xiaoni Zhang, Paul Hofman, Junji Uchino, Henry S. Park, Jose M. Pacheco, Fabrizio Tabbò, Mingyan Xu, Jiawei Dai, Kan He, Yang Yang, Caicun Zhou, written on behalf of the AME Lung Cancer Collaborative Group

**Affiliations:** ^1^ Department of Medical Oncology Shanghai Pulmonary Hospital and Thoracic Cancer Institute Tongji University School of Medicine No. 507, Zhengmin Road, Yangpu District Shanghai 200433 P. R. China; ^2^ HaploX Biotechnology, Co., Ltd. 8th floor, Auto Electric Power Building, Songpingshan Road, Nanshan District Shenzhen Guangdong 518057 P. R. China; ^3^ Department of Radiotherapy the eighth medical center of the Chinese PLA General Hospital No. 17, Heishanhu Road, Haidian District Beijing 100091 P. R. China; ^4^ Shenzhen HaploX Medical Laboratory 1106 South Block of Yuanxing Science and Technology Building, No. 1 Songpingshan Road, Xili Street, Nanshan District Shenzhen Guangdong 518057 P. R. China; ^5^ Laboratory of Clinical and Experimental Pathology FHU OncoAge BB‐0033‐00025 Pasteur Hospital University Côte d'Azur 30 avenue de la voie romaine, Nice cedex 01 Nice 06001 France; ^6^ Department of Pulmonary Medicine Kyoto Prefectural University of Medicine Kyoto 602‐8566 Japan; ^7^ Department of Therapeutic Radiology Yale University School of Medicine New Haven CT 06511 USA; ^8^ Thoracic Oncology Program Division of Medical Oncology Department of Internal Medicine University of Colorado Cancer Center Aurora CO 80045 USA; ^9^ Department of Oncology University of Turin San Luigi Hospital Orbassano Turin 10043 Italy; ^10^ SJTU‐Yale Joint Center for Biostatistics and Data Science Department of Bioinformatics and Biostatistics School of Life Sciences and Biotechnology Shanghai Jiao Tong University Shanghai 200240 China; ^11^ Department of Surgery, Shanghai Pulmonary Hospital Tongji University School of Medicine No. 507, Zhengmin Road, Yangpu District Shanghai 200433 P. R. China

**Keywords:** chemotherapy, lung cancer, NSCLC, SCLC, subtype, TMB

## Abstract

Evaluating the therapeutic response and survival of lung cancer patients receiving first‐line chemotherapy has always been difficult. Limited biomarkers for evaluation exist and as a result histology represents an empiric tool to guide therapeutic decision making. In this study, molecular signatures associated with response and long‐term survival of lung cancer patients receiving first‐line chemotherapy are discovered. Whole‐exome sequencing is performed on pretherapeutic tissue samples of 186 patients [145 non‐small cell lung cancer (NSCLC) and 41 small cell lung cancer (SCLC)]. On the basis of genomic alteration characteristics, NSCLC patients can be classified into four subtypes (C1–C4). The long‐term survival is similar among different subtypes. SCLC patients are also divided into four subtypes and significant difference in their progression free survival is revealed (*P* < 0.001). NSCLC patients can be divided into three subtypes (S1–S3) based on TMB. A trend of worse survival associated with higher TMB in subtype S3 than in S1+S2 is found. In contrast, no significant correlations between molecular subtype and therapeutic response are observed. In conclusion, this study identifies several molecular signatures associated with response and survival to first‐line chemotherapy in lung cancer.

## Graphical abstract

Evaluating the therapeutic response and survival of lung cancer patients receiving first‐line chemotherapy has always been difficult. In this study whole‐exome sequencing is performed on pretherapeutic tissue samples of 186 patients (145 non‐small cell lung cancer and 41 small cell lung cancer). Several molecular signatures associated with response and survival to first‐line chemotherapy in lung cancer are identified.

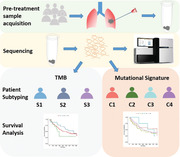

## 1 Introduction

It is widely accepted that the development of advanced non‐small cell lung cancer (NSCLC) relies on gene mutations and some of them act as drivers of neoplastic transformation.^[^ ^1^, ^2^, ^3^, ^4^ ^]^ Patients with mutations of epidermal growth factor receptor (*EGFR*) and translocations of anaplastic lymphoma kinase (*ALK*) can be treated with tyrosine kinase inhibitor (TKI)‐based target therapy whose efficacy has been fully demonstrated and many guidelines suggest the TKIs are the first line treatment for these patients.^[^ ^5^, ^6^, ^7^ ^]^

However, a substantial number of NSCLC patients lack oncogene drivers for which can be highly ineffective for targeted therapies.^[^ ^8^, ^9^, ^10^ ^]^ Chemotherapy still plays an important role in controlling tumor of these patients and is still one of the first‐line therapies for them.^[^ ^11^, ^12^ ^]^

Different chemotherapy regimens have shown differential effects on various types of lung cancer. For example, cisplatin/pemetrexed showed superior OS versus cisplatin/gemcitabine in patients with adenocarcinoma and large‐cell carcinoma histology, while there was a significant improvement in survival with cisplatin/gemcitabine versus cisplatin/pemetrexed for squamous cell carcinoma.^[^ ^13^ ^]^ Apart from histology, there is currently no effective method for evaluating patient response or survival to first‐line chemotherapy, thus therapeutic approaches rely mainly on physicians’ expertise and patients’ characteristics.

Since there are few reports investigating mutational characteristics and their correlation with chemotherapy outcomes in lung cancer patients with no TKI‐related driver gene mutations, we aimed to identify mutational characteristics associated with outcomes to first‐line chemotherapy. Substantial differences in the mutational spectrum and TMB were revealed. There was a strong correlation between molecular subtype and TMB with patients’ survival. Our study reveals molecular signatures that associate with survival and therapeutic response in lung cancer patients receiving first‐line chemotherapy.

## 2 Results

### 2.1 The Mutational Landscape of Lung Cancers without *EGFR* or *ALK* Driver Gene Alterations

**Figure 1:**
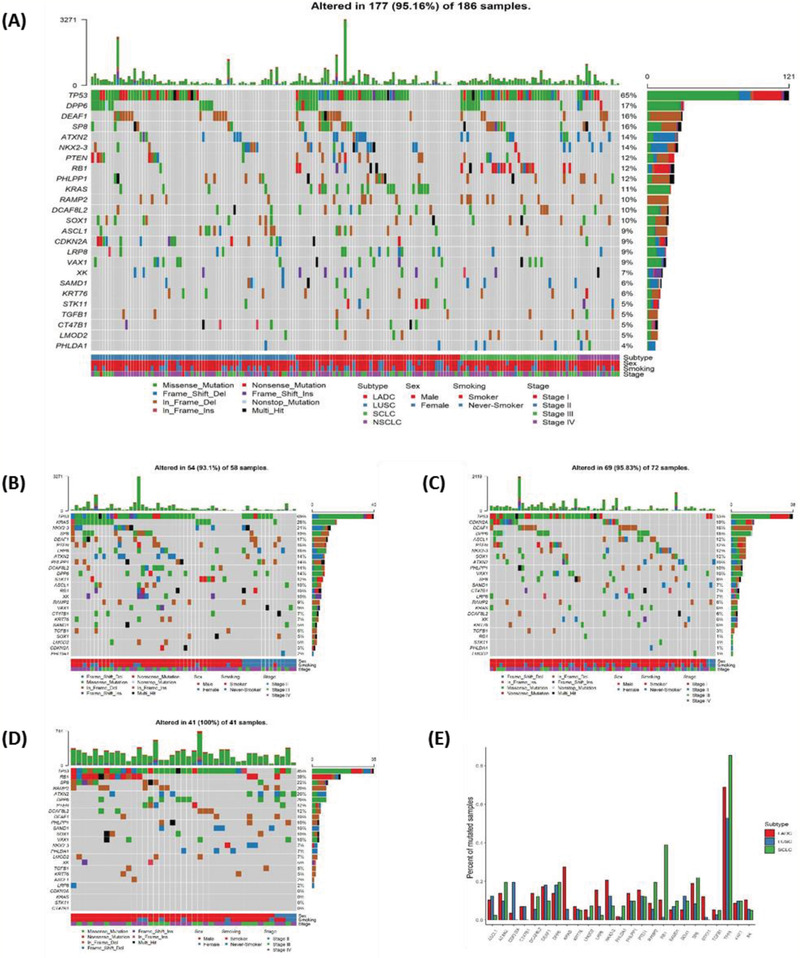
Mutational landscape in lung cancer without *EGFR* or *ALK* mutations. A) Mutational landscape of NSCLC and SCLC for all subjects involved in this study. Genes were aligned based on mutational frequency, and both SNV and INDEL mutations are shown. Clinicopathological factors, including pathological types, stages, sex, and smoking history are annotated; B) Full mutational landscape of all subjects with LADC; C) Full mutational landscape of all subjects with LUSC; D) Full mutational landscape of all subjects with SCLC; E) Comparison of the mutational frequency for commonly mutated genes in LADC, LUSC, and SCLC.

We evaluated the mutation spectrum and features of 186 lung cancer specimens with no mutations/translocations in *EGFR* or *ALK*. Our results showed significant differences in the distribution of sex, age, and smoking status of these patients compared with those with *EGFR* positive mutation or *ALK* fusion. In our study, 72.6% (135/186) of the patients had a history of smoking and 88.7% (165/186) of the patients were male (**Figure** **1**A). The proportion of male patients in the group without mutations/translocations in *EGFR* or *ALK* genes was higher compared to that of patients harboring a driver gene alteration. Adenocarcinoma (LADC), squamous cell carcinoma (LUSC), and small cell carcinoma (SCLC) accounted for 31.2% (58/186), 38.7% (72/186), and 22.0% (41/186), respectively. The proportion of LUSC was higher than that with *EGFR* positive mutation or *ALK* fusion (Figure 1A). *TP53*, *DPP6*, *DEAF1*, *SP8*, and *ATXN2* had the highest frequency of mutations in the whole cohort (Figure 1A). Different histological subtypes exhibited substantial variation in the mutational profile. For example, *KRAS* mutations were common in LADC (27.6%) while its frequency was significantly lower in LUSC (5.6%, *P* < 0.001, FDR = 0.010) and absent in SCLC (0.0%, *P* < 0.001, FDR = 0.009) (Figure 1B–E, Table S2, Supporting Information). *RB1* mutations were frequent in SCLC (39.0%) but much less frequent in LADC (10.3%, *P* < 0.001, FDR = 0.011) and LUSC (1.4%, *P* < 0.001, FDR < 0.001) (Figure 1B–E, Table S2, Supporting Information). High‐frequency mutation, *CDKN2A*, in LUSC (19.4%), was absent in SCLC (0.0%, *P* = 0.003, FDR = 0.032, Table S2, Supporting Information) (Figure 1C–E).

**Figure 2:**
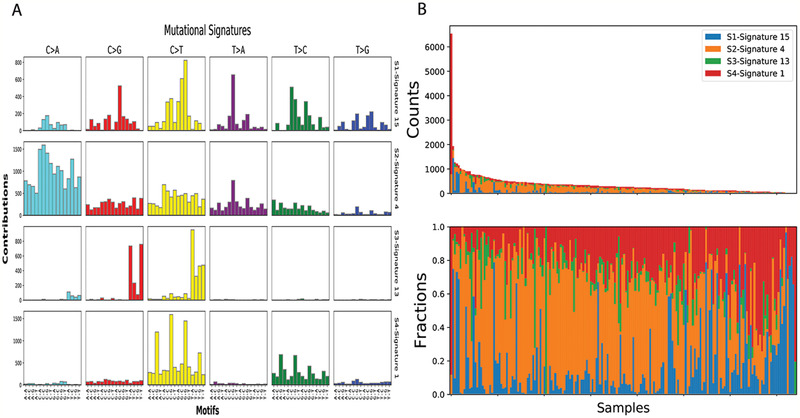
Mutation signature discovery by NMF for cancer tissues derived from lung cancer patients. A) Four mutational signature profiles across 96 mutation context. B) Fractions of mutational signatures across different samples.

By comparing mutational signatures of our data with 30 known COSMIC cancer signatures, we found four similar mutational signatures (**Figure** **2**A,B), which suggested that mutation in our data were similar to those caused by age, smoking, function of APOBEC family, and dysfunction of DNA mismatch repair.

Unsupervised clustering analysis was performed later. Both NSCLC and SCLC can be subdivided into four groups according to their mutation signature (C1–C4 for NSCLC, C5–C8 for SCLC). **Figure** **3**A shows the mutational signatures for each of these subtypes. C1 of NSCLC and C5 of SCLC had enriched mutational signature 15 whose potential cause was dysfunction of DNA mismatch repair. C2 of NSCLC and C8 of SCLC had enriched mutational signature 1 which was correlated with age. C3 of NSCLC and C6 of SCLC had enriched mutational signature 13 which might have relationship with the activity of enzymes of APOBEC family. C4 of NSCLC and C7 of SCLC had enriched mutational signature 4 which might be caused by smoking. We further analyzed the proportion of patient with smoking history in different subtypes. In NSCLC, we found patients with smoking history were more likely to have mutational signature 4 (*P* = 0.006). Although C7 subtype of SCLC also had enriched mutational signature 4, we did not find the proportion of patient with smoking history was different between C7 subtype and non‐C7 subtype (*P* = 0.807). Figure 3B shows the correlation of subtype with the specific gene mutations in NSCLC. And we found that mutational frequency of *TP53* was significantly lower in C1 subtype (C1 vs C2, *P* < 0.001, FDR = 0.0049; C1 vs C3, *P* < 0.001, FDR = 0.0049; C1 vs C4, *P* < 0.001, FDR < 0.001). Figure 3C compares the TMB of the four NSCLC molecular subtypes: the TMB of C3 and C4 subtypes was high, while the TMB of the other two subtypes was low, with C2 subtype being the lowest.

Figure 3Molecular subtyping and evaluation of long‐term survival in lung cancer patients without driver gene mutations. A) Mutational signatures for the four NSCLC molecular subtypes (C1–C4) and four SCLC subtypes (C5–C8). B) The association between NSCLC molecular subtype and specific gene mutations. C) Comparison of TMB for subtypes C1–C4 of NSCLC. D) Overall survival for each NSCLC molecular subtype (*P* = 0.780). E) Progression free survival for each NSCLC molecular subtype (*P* = 0.800). F) Overall survival for each SCLC molecular subtype (*P* = 0.770). G) Comparison of overall survival between subtype C7 and C8 of SCLC (*P* = 0.830). H) Progression free survival for each SCLC molecular subtype (*P* < 0.001). I) Comparison of progression free survival between subtype C7 and C8 of SCLC (*P* < 0.001).

**Figure:**
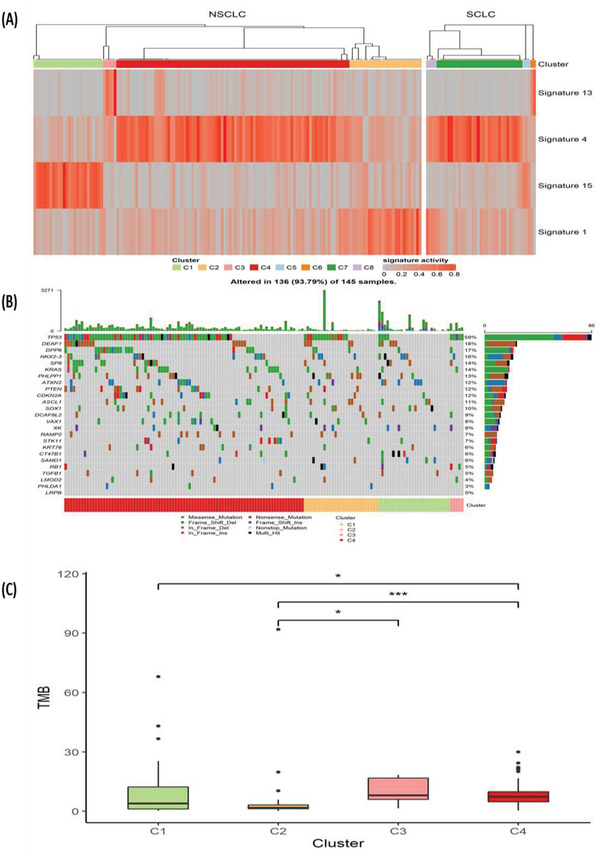

**Figure:**
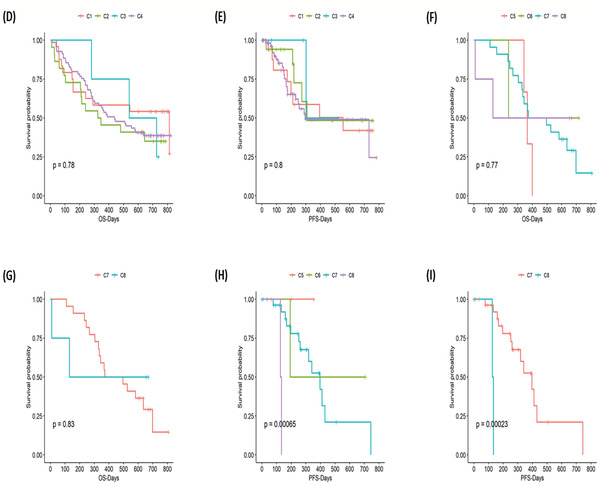

### 2.2 Evaluation of Therapeutic Response and Prognosis by Molecular Subtyping

We further studied the correlation between molecular subtyping and the therapeutic response to the first‐line chemotherapy. **Table** **1** shows no significant differences among the four molecular subtypes with regards to partial response (PR) or non‐PR. The *p* value for NSCLC C1–C4 was 1.000 (Fisher = 0.432), and for SCLC C5–C8 was 0.200 (Fisher = 3.978) for SCLC.

**Table 1:** The number and ratio of subjects with PR or non‐PR

	NSCLC			SCLC		
	PR	Non‐PR	PR [%]	PR	Non‐PR	PR [%]
C1	3	23	11.5	–	–	–
C2	4	23	14.8	–	–	–
C3	0	5	0.0	–	–	–
C4	12	75	13.8	–	–	–
C5	–	–	–	2	1	66.7
C6	–	–	–	1	1	50.0
C7	–	–	–	9	23	28.1
C8	–	–	–	0	4	0.0

By following all patients for up to 824 d (median 441 d), we investigated whether molecular subtyping can evaluate the survival of lung cancer patients without targetable oncogene drivers. Figure 3D,E shows the OS and PFS for the four NSCLC molecular subtypes, respectively. The one‐year OS rate for the NSCLC C1–C4 subtypes was 58.3%, 45.5%, 75.0%, and 53.6%, respectively. And we did not find the significant difference of survival among different molecular subtypes of NSCLC. The OS and PFS for SCLC molecular subtypes are shown in Figure 3F and 3H, respectively. The one‐year OS rate for SCLC C5–C8 subtypes was 33.3%, 50.0%, 59.1%, and 50.0%, respectively. In the current data, we could find the PFS (*P* < 0.001) were significantly different among SCLC subtypes. C8 subtype showed the worst survival in PFS, but this might be caused by the extremely small size of this cluster. Further analysis also found C7 subtype had a better PFS than C8 subtype in SCLC patients (*P* < 0.001) but this difference was not found in OS (*P* = 0.83).

### 2.3 Patients’ Subtyping Based on TMB

The distribution of TMB had not been fully characterized in Chinese lung cancer patients with no *EGFR* or *ALK* mutations. **Figure** **4**A shows the distribution of TMB from high to low in LADC, LUSC, and SCLC. It could be seen that TMB distributed in a wide range, with the majority of patients having relatively low TMB (<10 muts/Mb), but a small proportion of patients exhibiting a high level of TMB (>10 muts/Mb). We further studied The TMB within different lung cancer histologies. TMB was similar between NSCLC and SCLC as well as between LUSC and LUAD (Figure 4A). TMB distribution was shown in Figure 4B and most patients’ TMB level was lower than 10 mutations/Mb.

Figure 4TMB distribution, subtyping, and its correlation with survival. A) Distribution of TMB from high to low for LADC, LUSC, and SCLC patients. No significant difference was observed between NSCLC and SCLC, or between LADC and LUSC. B) Distribution of TMB in NSCLC: three subtypes based on TMB level were discovered and named S1, S2, and S3, respectively. C) Mutational frequency for cancer‐related genes within NSCLC TMB subtype S1, S2, and S3. D) Mutational landscape of cancer‐related genes based on NSCLC TMB subtyping, and the genes were ranked by mutational frequency. E) Mutational landscape of most frequently mutated genes based on NSCLC TMB subtyping, and the genes were ranked by mutational frequency. F) Mutational frequency for most frequently mutated genes within NSCLC TMB subtype S1, S2, and S3. G) The Kaplan–Meier survival analysis for OS of NSCLC TMB subtypes S1–S3 (*P* = 0.46). H) Comparison of OS between NSCLC TMB subtype S2 and S3 (*P* = 0.17). I) Comparison of OS between NSCLC TMB subtype S1+S2 and S3 (*P* = 0.29). J) The Kaplan–Meier survival analysis for PFS of NSCLC TMB subtypes S1–S3 (*P* = 0.26). K) Comparison of PFS between NSCLC TMB subtype S2 and S3 (*P* = 0.44). L) Comparison of PFS between NSCLC TMB subtype S1+S2 and S3 (*P* = 0.12).

**Figure:**
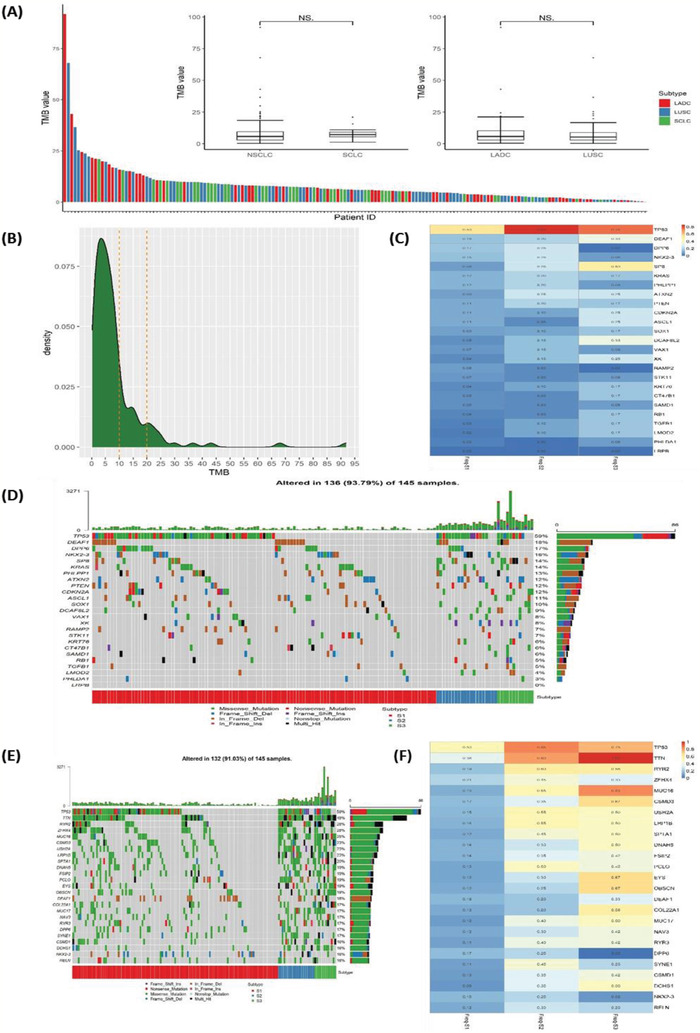

**Figure:**
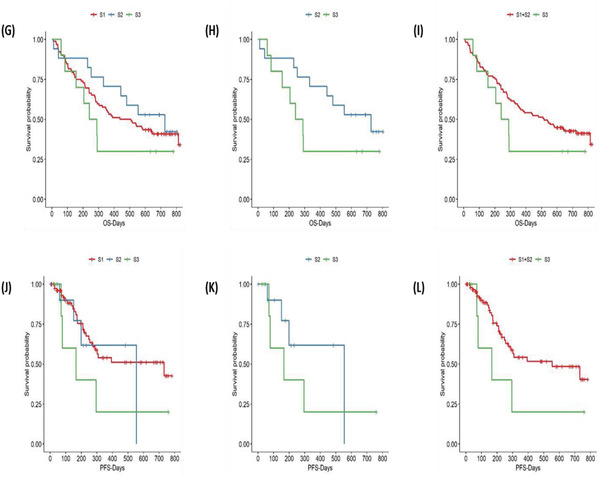

We further analyzed The distribution of TMB by dividing it into three components (S1, S2, and S3) based on the level of TMB. S1 represented the low TMB group (TMB ≤ 10 mutations/Mb), and it accounted for more than half of the patients, while the S2 represented the medium TMB group (10 mutations/Mb < TMB ≤ 20 mutations/Mb). The S3 group represented the highest TMB group (TMB > 20 mutations/Mb), with the least number of patients (Figure 4D). Among these three TMB subtypes, the high‐frequency mutational pattern of cancer‐related genes identified from our data through MutSigCV was investigated (Figure 4C). The mutational frequency for most genes in S3 was higher than that in S1 and S2, and the frequency of the *SP8* mutations in S3 was significantly higher than that in S1 (*P* < 0.001, FDR = 0.008), which had a strong correlation with high TMB. The mutation spectrum in Figure 4D also confirmed the above observation. We also compared the mutation frequency of the most frequently mutated genes identified from our data among three subtypes (Figure 4E). The mutational frequency for each gene in S2 and S3 was higher than that in S1, and the frequency of the *TP53* and *TTN* mutations in S2 and S3 was higher than that in S1 (Figure 4F).

We analyzed The correlation between TMB and therapeutic response to first‐line chemotherapy in NSCLC. **Table** **2** demonstrates no significant difference in response between the three TMB subtypes (*P* = 1.000).

**Table 2:** The number and ratio of subjects with PR or non‐PR based on NSCLC TMB subtyping

	PR	Non‐PR	PR [%]
S1	16	97	14.2
S2	2	18	10.0
S3	1	11	8.3

We followed patients for up to 824 d (median 441 d) and analyzed the difference in PFS and OS based on TMB subtype within NSCLC. The S1, S2, and S3 subtypes exhibited a one‐year OS rate of 53.3%, 70.6%, and 30.0%, respectively. It could be observed from Figure 4G that no significant differences existed among S1–S3. There was a trend that the OS and PFS of S3 would be worse than S2 and S1+S2 subtype (Figure 4H,I,K,L), but it was not significant.

**Figure 5:**
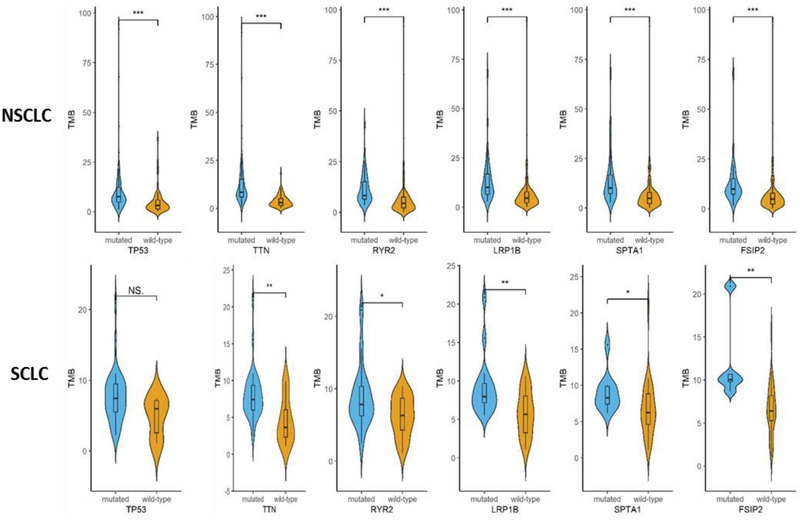
Correlation between high‐frequency mutations and TMB. A significant difference or trend in differences for TMB was observed between subjects with mutated genes (including *TP53*, *TTN*, *RYR2*, *LRP1B*, *FSIP2*, and *SPTA1*) and those subjects without mutated genes. *P < 0.05; **P < 0.01; ***P < 0.001. NS: not significant.

The relationship between representative high‐frequency mutated genes and the level of TMB was also investigated. **Figure** **5** indicated that the TMB of patients with *TP53*, *TTN*, *RYR2*, *LRP1B*, *FSIP2*, and *SPTA1* mutations was significantly higher than that of patients without these mutations for both NSCLC and SCLC. Although there was no statistically significant difference for *TP53*, the analysis confirmed a similar trend in SCLC. These high‐frequency mutations are closely related to the higher TMB (Figure 5). Moreover, observation on correlation between high‐frequency mutations and TMB also suggested that TMB was not correlated with mutations of individual genes but multiple genes.

## 3 Discussion

Our study is the first to study the lung cancer patients’ mutation spectrum in the Chinese population without *EGFR* or *ALK* driver gene mutations. Many patients lacking targetable oncogene drivers receive chemo‐immunotherapy regimens as initial treatment. Unfortunately, there is little data on biomarkers that associate with efficacy of first‐line chemotherapy based regimens. Therefore, it is important to study the mutational spectrum of these patients to understand molecular subtyping and the relationship with response and prognosis. In this study, we found that the frequency of *TP53* and *DPP6* gene mutations in all lung cancer were higher than that of other genes, and the frequency of *TP53* mutations in SCLC was particularly high (85.4%) and significantly higher than that in LUSC. The mutation rate of *RB1* was 39.0% in SCLC, while it was only 10.3% and 1.4% in LADC and LUSC, respectively. In addition, *NKX2‐3*, *DEAF1*, and *SP8* were found to have high mutation frequencies in LADC, LUSC, and SCLC, respectively.

Our study found that the proportion of patients in different pathological types or genders was different from all previous reports in Chinese lung cancer. First, LADC was the dominant type in previous reports, followed by LUSC and SCLC.^[^ ^16^ ^]^ However, the proportion of LUSC was higher than that of LADC in our study, indicating an alteration in histologic distribution within patients lacking a targetable oncogene driver. This discrepancy with previous reports could be due to the higher ratio of patients in LADC with targetable oncogene drivers. In addition, 88.7% (165/186) of the patients were male, which was higher than that of some reports in China.^[^ ^16^ ^]^ This may be due to the exclusion of *EGFR* or *ALK*‐related driver gene mutations, as a considerable proportion of patients with TKI‐related driver gene mutations were nonsmoking patients. Therefore, the therapeutic response and long‐term prognosis of LUSC and male patients with no *EGFR* or *ALK*‐related driver gene mutations are worth further investigation.

NSCLC and SCLC were divided into four molecular subtypes according to their mutational signatures. In NSCLC, we evaluated the relationship between subtypes and TMB. The difference in TMB among various subtypes reflected the difference in mutational frequency. The TMB subtype in NSCLC did not affect the therapeutic response to first‐line chemotherapy. However, TMB might affect the response to second‐line or multiline immunotherapy, as TMB is a potential predictor for response to immunotherapy.

In this study, the distribution of TMB in lung cancer patients was evaluated by stratification. The high, middle, and low TMB subtypes were determined based on the TMB distribution curve (S1–S3). The mean value of TMB in SCLC was higher than that in NSCLC but no statistic difference reached. And there was also no difference in TMB between LADC and LUSC. Meanwhile, a recent study suggested that one should be cautious when interpreting TMB from NGS panel‐based methods using the correlation or the coefficient of determination, especially for patients with TMB of 5 to 25 muts/Mb.^[^ ^17^ ^]^ Although using the targeted sequencing panels was possible and predictive of response to immune checkpoint inhibitor (ICI) treatment, the agreement assessed between panels within the above TMB scope was low.^[^ ^17^ ^]^ Therefore, WES may be better than panels in assessing the TMB with less across‐panel variation.

### 3.1 Molecular Subtype and TMB Subtype Associate with Survival but Not Response to First‐Line Chemotherapy

An interesting finding of this study was that molecular subtype could potentially evaluate the survival of SCLC patients when these patients were treated with first‐line chemotherapy. The SCLC C8 subtype patients would have a worse PFS of first chemotherapy than those patients who were defined as C7 subtype. Although there was no statistical difference, in TMB subtyping, S3 with the highest TMB showed a trend of worse OS and PFS. These observations strongly suggested that high TMB might be a potential indicator of worse survival in NSCLC patients treated with first‐line chemotherapy. For the first time our study identified possible indicators of survival to first‐line chemotherapy based regimens. In addition, previous studies have suggested that TMB is an indicator of the response of immunotherapy,^[^ ^17^ ^]^ while our study suggested that TMB, rather than therapeutic strategy, might be the key factor in evaluating patients’ benefit. In recent reports, immunotherapy combined with radiotherapy, targeted therapy, chemotherapy, or anti‐endothelial growth factor (VEGF) therapies has shown that TMB cannot effectively stratify the response or survival of patients. This may be due to the enhanced efficacy of combination therapy, which diminished the role of TMB on stratification.^[^ ^18^, ^19^ ^]^

In this study, the molecular subtypes we identified were found to associate with survival to first‐line chemotherapy based regimens. As mentioned in the Introduction, the standard of care in first‐line treatment has now changed to immunotherapy or chemo‐immunotherapy. It is unknown whether these molecular subtypes would associate with survival to immunotherapy or chemo‐immunotherapy regimens. It appeared that the molecular subtypes in our study were not related to certain gene mutations but the pattern of mutations involving multiple genes, which is quite different to the molecular alterations when the driver genes were not excluded.^[^ ^5^, ^20^, ^21^ ^]^ Therefore, further investigation in this regard is warranted. Furthermore, since the molecular subtypes described were associated with survival to first‐line chemotherapy, it is possible they may similarly associate with survival to subsequent line chemotherapies (e.g., docetaxel or gemcitabine in NSCLC or topotecan in SCLC). For those who have no chance for immunotherapy or chemo‐immunotherapy, it would be valuable to determine whether the molecular subtypes also evaluate the survival of multilines of chemotherapies. Similarly, we studied the advanced or unresectable lung cancer patients treated with initial platinum based doublet chemotherapy, whether the molecular subtypes are associated with benefit or lack of adjuvant therapies on early‐stage patients who received surgery is also worth further investigation. It is also worth investigating whether certain molecular subtypes have a differential response to specific chemotherapies. Finally, our findings should be validated in a larger cohort of patients with longer follow‐up time, especially for SCLC patients.

We also found that molecular subtype and TMB subtype could not predict the response to first‐line chemotherapy, indicating that they were not indicators for the extent of tumor remission. Although the correlation between response and survival is certain, our study suggested that if the response of patients were mainly PR and SD, the factors determining the survival are not confined to therapeutic response.

In this study, there were 12 patients with stage I or II who were not surgical candidates and 174 with stage III or IV unresectable disease. Given the small percentage of early stage disease (stage I and II), the differences in stage distribution unlikely affected association between outcomes observed for molecular subtypes, TMB subtypes and/or specific mutations, since no gross imbalances in molecular subtype were found for the 12 patients.

It should be noted that the mutational spectrum of a tumor may change with therapy, and this could happen after treatment with chemotherapy, immunotherapy, or chemo‐immunotherapy. Therapeutic selection of resistant clones and tumor evolution may change the tumor sensitivity to therapy sooner or later. Thus, it is possible that the initial diagnostic molecular testing may not always be predicative of subsequent line therapy responses, and therefore re‐biopsy is helpful upon the development of resistance.

Some limitations to this study, mainly involving the follow‐up time and the response data, should also be addressed. The follow‐up time of 824 d might not have been sufficiently extensive to examine the long‐term survival, and ideally, more than five‐year follow‐up should be performed for all patients. Meanwhile, only a few patients experienced PD, therefore it would be difficult to see any significant result if PD was regarded as one group, instead, we divided all patients into the PR group or the non‐PR group (including SD and PD). A further weakness of this study was the relatively small sample size of patients. Some patients’ survival state missing was another limitation. This was due to many reasons, including loss of follow‐up, patient transfer to another hospital, treatment in local hospitals, or no treatment.

## 4 Experimental Section

##### Ethical Approval by Participating Hospitals

All experimental plans and protocols for the study were submitted to the ethics/licensing committee of the Shanghai Pulmonary Hospital for review and confirmation. Approval for the study (No. FK18‐203) was received from the ethics committee of Shanghai Pulmonary Hospital. Written informed consent was obtained from all participating patients. All experiments, methods, procedures, and personnel training were conducted following relevant guidelines and regulations of the participating hospital and laboratories.

##### Study Design, Patients, and Samples Collection

The study was designed and implemented in Shanghai Pulmonary Hospital. Cancer tissue and blood samples were collected prospectively. The study was designed to include NSCLC patients without *EGFR* (SNV, INDEL, Amp) and *ALK* (SNV, INDEL, fusion). PCR assays targeting *EGFR* and *ALK* were performed for all available patients before any therapy. As a result, samples from 186 qualifying lung cancer patients were obtained, including 145 NSCLC and 41 SCLC patients, and clinic‐pathological features were acquired (Table S1, Supporting Information). Collected samples include fresh or frozen specimens from needle biopsies and blood samples were obtained at the same time. All technicians were blinded to the subjects’ clinical information. Confirmation of pathological types was based on diagnosis from imaging evaluations and subsequent pathological examinations. None of the subjects received chemotherapy, radiotherapy, targeted therapy, or immunotherapy before tissue or blood samples were collected. In the 186 patients, 174 patients were advanced lung cancer. The somatic sequencing data presented in this study were from lung tumor tissue DNA and germline sequencing data were obtained from the corresponding white blood cell genomic DNA.

##### *EGFR* Mutation Status and *ALK* Rearrangement Examination

Genomic DNA or RNA was extracted from tissue samples according to the manufacturer's protocol. The extracted RNA would be reversed transcripted to cDNA for latter polymerase chain reaction (PCR) amplification and examination. *EGFR* mutations in exons 18‐21 and *EML4‐ALK* rearrangement were detected by AmoyDx *EGFR* 29 Mutations Detection Kit (Amoy, Xiamen, China) and AmoyDx *EML4‐ALK* Fusion Gene Detection Kit (Amoy, Xiamen, China) separately according to the protocols. Patients with 19del, L858R, T790M, 20ins, G719X (X = A, C, or S), S768I, or L861Q would be classified as *EGFR* mutated. Patients with positive *EML4‐ALK* fusion were classified as *ALK* rearranged. The cutoff ΔCt values for 19del, L858R, T790M, 20ins, G719X, S768I, and L861Q were 13, 14, 12.5, 13, 13, 12, and 12, respectively. And if any FAM Ct value of EA Fusion Gene Reaction Mix < 30, the sample would be determined as *EML4‐ALK* fusion positive.

##### Sample Preparation for Targeted Next Generation Sequencing (NGS) and Whole‐Exome Sequencing (WES) Data Processing

DNA was extracted from tissue samples using the QIAamp DNA Tissue Kit (QIAGEN, Valencia, CA) following the manufacturer's instructions. For blood samples, 10 mL of blood was collected in EDTA tubes and centrifuged at 1600× *g* for 10 min (4 °C) within 2 h of collection. The peripheral blood lymphocyte (PBL) debris was stored at −20 °C for later use. The supernatants were further centrifuged at 10 000× *g* for 10 min (4 °C), and plasma was harvested and stored at −80 °C for later use. DNA was extracted from PBLs using the RelaxGene Blood DNA system (Tiangen Biotech Co., Ltd., Beijing, China). Both cancer tissue and white blood cell genomic DNA were quantified with the Qubit 2.0 Fluorometer and the Qubit dsDNA HS assay kit (Thermo Fisher Scientific, Inc., Waltham, MA) according to manufacturer's instructions. In brief, fragmented genomic DNA underwent end‐repairing, A‐tailing and ligation with indexed adapters sequentially, followed by size selection using Agencourt AMPure XP beads (Beckman Coulter Inc., Brea, CA) and DNA fragments were used for library construction with the KAPA Library Preparation kit (Kapa Biosystems, Inc., Wilmington, MA) according to the manufacturer's protocol. Hybridization‐based target enrichment was conducted with the HaploX WESPlus gene panel (an upgraded version of the standard WES, HaploX Biotechnology) for cancer tissue sequencing. Seven to eight PCR cycles, depending on the amount of DNA input, were performed on Pre‐capture ligation mediated PCR (Pre‐LM‐PCR) Oligos (Kapa Biosystems, Inc.) in 50 µL reactions. The DNA sequencing was then performed on the Illumina Novaseq 6000 system according to the manufacturer's instructions at an average depth of 500×.

For somatic mutation detection, the Genome Analysis Toolkit (GATK, Version 4.1.7.0) best practice workflow was followed for somatic short variant discovery. Sequencing data were aligned to the hg19 genome (GRch37) using Burrows‐Wheeler Aligner (BWA, Version: 0.7.17‐r1198) with default settings. Duplicated reads were subsequently marked and removed using the GATK Picard tool. After the base quality score recalibration using BaseRecalibrator and ApplyBQSR functions of GATK, SNVs and INDELs were called from tumor and matched‐normal pairs using Mutect2 from GATK. MutSigCV (Version: 1.41) was used to determine significantly mutated gene with a *q* value below 0.05.

##### Mutational Signature Analysis

The exaction of the mutational signatures in the tumor samples was performed with SignatureAnalyzer. Non‐negative matrix factorization algorithm (NMF) was applied for mutational signature analysis. The mutational signatures detected in the samples were compared to 30 known COSMIC cancer signatures.

##### Cluster Analysis

The activity matrix of each mutational signature contributing to tumor samples was determined. Then, an unsupervised tool ConsensusClusterPlus was used for consensus clustering. 80% item resampling (pItem), 10 resamplings (reps), and Pearson correlation distances (distance) as settings of the ConsensusClusterPlus were selected. Eventually, four clusters of NSCLC samples and SCLC samples were determined.

##### Statistics, Data Analysis, Calculation of Somatic TMB, and Molecular and TMB Subtyping

Statistical analysis was performed and figures were plotted with GraphPad Prism 5.0 software (GraphPad Software, Inc., La Jolla, CA). Student's *t*‐test or nonparametric test was performed when two groups were compared, while ANOVA and post hoc tests were performed when three or more groups were compared. Chi‐square test and Fisher test were performed when rate or percentage was compared for significance. And the Benjamini–Hochberg procedure was used to correct *P* values for multiple hypotheses testing when appropriate. Figures were made with PRISM 5.0 or the R software (https://www.r-project.org/). * represents *P* < 0.05 (significant), ** represents *P* < 0.01 (highly significant), and *** represents *P* < 0.001 (very highly significant). TMB was calculated by dividing the total number of tissue nonsynonymous SNV and INDEL variations (allele frequency > 5%) by the full length of the WES panel. Molecular subtyping and TMB subtyping were performed based on modified methods from previous reports^[^ ^14^, ^15^ ^]^ and figures were made with the R software tools (https://github.com/raerose01/deconstructSigs; https://bioconductor.org/packages/release/bioc/html/ConsensusClusterPlus.html).

## Conflict of Interest

The authors declare no conflict of interest.

## Supporting information

Supporting Information
