# Supplementary material for: Mutational Profile Evaluates Response and Survival to First‐Line Chemotherapy in Lung Cancer
Source: Adv Sci (Weinh). 2020 Dec 30;8(4):2003263. doi: 10.1002/advs.202003263 (PMC7887584; doi:10.1002/advs.202003263)
Supplement: Supplementary file 1 — Supporting Information [file ADVS-8-2003263-s001.pdf]

WILEY-VCH

© 2020 Wiley-VCH GmbH

ADVANCED SCIENCE
Open Access

Supporting Information

for *Adv. Sci.,* DOI: 10.1002/advs.202003263

Mutational Profile Evaluates Response and Survival to First-line Chemotherapy in Lung Cancer

Yayi He, Lele Song, Hao Wang, Peixin Chen, Yu Liu, Hui Sun, Xiaobin Li, Shiying Dang, Guifeng Liu, Xinyi Liu, Shifu Chen, Xiaoni Zhang, Paul Hofman, Junji Uchino, Henry S. Park, Jose M. Pacheco, Fabrizio Tabbò, Mingyan Xu, Jiawei Dai, Kan He, Yang Yang, Caicun Zhou,* written on behalf of the AME Lung Cancer Collaborative Group*

Supplementary Table 1. Clinicopathological and demographic information of all patient involved in this study

		Number of patients
**Total**		186
**Median Age, years (range)**		67（22-84）
**Sex**		
	Male	165
	Female	23
**Histology**		
	LADC	58
	LUSC	72
	NSCLC（uncertain）	15
	SCLC	41
**Smoking status**		
	Ever	135
	Never	51
**Stage**		
	I	4
	II	8
	III	64
	IV	110
**Best overall response**		

PR	30
SD	66
PD	16
uncertain	74

NSCLC=non-small cell lung cancer; SCLC=small cell lung cancer; LADC=lung adenocarcinoma; LUSC=lung squamous cell carcinoma; PR=partial response: SD=stable disease; PD=progressed disease.

Supplementary Table 2 Gene mutation frequencies among three pathological subtypes

Gene	Subtype 1	Subtype 2	mutation frequency in subtype 1	mutation frequency in subtype 2	P_value	FDR
RB1	LUSC	SCLC	1.4%	39.0%	7.42E-08	5.57E-06
KRAS	LADC	SCLC	27.6%	0.0%	0.00024	0.008991
TP53	LUSC	SCLC	52.8%	85.4%	0.000496	0.010101
KRAS	LADC	LUSC	27.6%	5.6%	0.000539	0.010101
RB1	LADC	SCLC	10.3%	39.0%	0.000722	0.010835
CDKN2	LUSC	SCLC	19.4%	0.0%	0.00255	0.03195

A	7	8
